# IgG antibodies to synthetic GPI are biomarkers of immune-status to both *Plasmodium falciparum* and *Plasmodium vivax* malaria in young children

**DOI:** 10.1186/s12936-017-2042-2

**Published:** 2017-09-25

**Authors:** Camila T. França, Connie S. N. Li Wai Suen, Amandine Carmagnac, Enmoore Lin, Benson Kiniboro, Peter Siba, Louis Schofield, Ivo Mueller

**Affiliations:** 1 Population Health and Immunity Division, Walter and Eliza Hall Institute, Parkville, VIC Australia; 2 Department of Medical Biology, University of Melbourne, Melbourne, VIC Australia; 3 Infection and Immunity Division, Walter and Eliza Hall Institute, Parkville, VIC Australia; 4 Malaria Immuno-Epidemiology Unit, PNG Institute of Medical Research, Madang, Madang Province Papua New Guinea; 5 Australian Institute of Tropical Health and Medicine, James Cook University, Townsville, QLD Australia; 6 Malaria Parasites & Hosts Unit, Department of Parasites & Insect Vectors, Institut Pasteur, Paris, France; 7 Barcelona Institute of Global Health (ISGLOBAL), Barcelona, Spain

**Keywords:** *Plasmodium falciparum*, *Plasmodium vivax*, Malaria elimination, IgG antibody, Biomarker of exposure, GPI, Glycosylphosphatidylinositol, Clinical malaria, Protection, Exposure

## Abstract

**Background:**

Further reduction in malaria prevalence and its eventual elimination would be greatly facilitated by the development of biomarkers of exposure and/or acquired immunity to malaria, as well as the deployment of effective vaccines against *Plasmodium falciparum* and *Plasmodium vivax.* A better understanding of the acquisition of immunity in naturally-exposed populations is essential for the identification of antigens useful as biomarkers, as well as to inform rational vaccine development.

**Methods:**

ELISA was used to measure total IgG to a synthetic form of glycosylphosphatidylinositol from *P. falciparum* (*Pf*GPI) in a cohort of 1–3 years old Papua New Guinea children with well-characterized individual differences in exposure to *P. falciparum* and *P. vivax* blood-stage infections. The relationship between IgG levels to *Pf*GPI and measures of recent and past exposure to *P. falciparum* and *P. vivax* infections was investigated, as well as the association between antibody levels and prospective risk of clinical malaria over 16 months of follow-up.

**Results:**

Total IgG levels to *Pf*GPI were low in the young children tested. Antibody levels were higher in the presence of *P. falciparum* or *P. vivax* infections, but short-lived. High IgG levels were associated with higher risk of *P. falciparum* malaria (IRR 1.33–1.66, P = 0.008–0.027), suggesting that they are biomarkers of increased exposure to *P. falciparum* infections. Given the cross-reactive nature of antibodies to *Pf*GPI, high IgG levels were also associated with reduced risk of *P. vivax* malaria (IRR 0.65–0.67, P = 0.039–0.044), indicating that these antibodies are also markers of acquired immunity to *P. vivax*.

**Conclusions:**

This study highlights that in young children, IgG to *Pf*GPI might be a useful marker of immune-status to both *P. falciparum* and *P. vivax* infections, and potentially useful to help malaria control programs to identify populations at-risk. Further functional studies are necessary to confirm the potential of *Pf*GPI as a target for vaccine development.

**Electronic supplementary material:**

The online version of this article (doi:10.1186/s12936-017-2042-2) contains supplementary material, which is available to authorized users.

## Background

Despite several countries having reduced malaria incidence by more than 75%, and a reduction in mortality by 48% globally, more than 3 billion people are still at risk of contracting malaria and some 438,000 deaths still occur every year [1]. Current malaria control and elimination efforts would be greatly enhanced by the development of novel and more sensitive surveillance tools. For instance, serological markers that can be used to estimate exposure to malaria parasites and/or indicate a person’s immune status would help to identify populations at risk, and to direct resources to areas in more need [2–4]. Additionally, the development and deployment of highly efficacious vaccines against the two major malaria parasites, *Plasmodium falciparum* and *Plasmodium vivax,* would certainly accelerate malaria elimination [2, 5].

Identifying optimal antigenic targets for evaluating exposure or for vaccine development, however, remains a huge challenge due to the complexity of malaria parasites biology and epidemiology [6]. As the dynamics of antibody acquisition and maintenance vary based on exposure intensity, which serologic markers are informative of exposure or immunity is likely to differ by age group and transmission setting [4, 7, 8]. A better understanding of the human immune responses to malaria parasites is thus essential for biomarker discovery, and very useful in guiding rational vaccine design [4, 7, 8]. To date, relatively little is known about the early acquisition and role of anti-*Plasmodium* spp. antibodies in young children, how such responses compare to responses in older children/adults, or those from different transmission intensity areas [7–11]. The investigation of antigenic targets and their potential as vaccine candidates or biomarkers of exposure in naturally exposed populations has been mainly restricted to *P. falciparum* and very few *P. vivax* merozoite proteins [7–11].

In malaria parasites, glycosylphosphatidylinositol (GPI) is a glycolipid highly conserved across different species [12]. In *Plasmodium* spp., GPI can be found both free and as an anchor sustaining many proteins on the parasite’s membrane, including merozoite surface and rhoptry proteins, as well as many other vaccine candidates and proteins of unknown function [12]. In humans, GPI is known to induce strong humoral response, promote the expression of genes of pro-inflammatory compounds (including tumour-necrosis factor (TNF), interleukin-1 [IL-1] and IL-12), nitric oxide, and adhesion molecules on the surface of the vascular endothelium, which can be recognized by *P. falciparum* erythrocyte membrane protein 1 (*Pf*EMP1), contributing to the development of anaemia and severe malaria [13, 14].

It has been consistently demonstrated that GPIs purified from *P. falciparum* are recognized by plasma/serum from people living in malaria-endemic areas however, the quality of GPIs purified from *P. falciparum* might have led to controversial results [15, 16]. Cross-reactivity between antibodies raised against *P. falciparum* GPI and *P. vivax* is expected, as despite having a high complexity that allows various chemical modifications and high functional diversity, the core of the GPI glycan structure is evolutionary highly conserved in different species [17]. Only limited structural variability (in fatty-acid composition or glycosylation) or antigenic variation have been described [18–21] in comparison to the many allelic polymorphisms identified in merozoite surface proteins [22–24], and the consequent high antigenic variation [25–27].

To date, the association between the levels of antibodies to GPI and the risk of malaria clinical disease remains poorly explored. To address this gap, this study aimed to measure total IgG levels to a synthetic glycan corresponding to *P. falciparum* GPI (*Pf*GPI) in a cohort of children aged 1–3 years from Papua New Guinea (PNG), exploring the associations between antibody levels and prospective risk of malaria. Individual differences in exposure to *Plasmodium* spp. blood-stage infections have been well characterized by molecular genotyping [28, 29], and children have been shown to had acquired immunity to *P. vivax,* but no yet to *P. falciparum* [28–30]. The potential use of IgG to *Pf*GPI as a serological biomarker of immune status to both *P. falciparum* and *P. vivax* parasites was investigated.

## Methods

### Antigen

The synthetic glycan *Pf*GPI described by Schofield et al. [31] was used. As the glycan was conjugated to bovine serum albumin (BSA), BSA alone was included as a control.

### Study samples

Antibody reactivity to *Pf*GPI in naturally exposed individuals was assessed in samples from a longitudinal cohort of 264 children (1–3 years old) undertaken in Ilaita, East Sepik Province, PNG [30]. Children were enrolled between March and September 2006, and followed for up to 16 months. Blood samples were collected every 8 weeks and at episodes of febrile illness. All *P. falciparum* and *P. vivax* infections were genotyped, allowing the determination of the incidence of genetically distinct blood-stage infections acquired during follow-up (i.e. the molecular force of blood-stage infections, molFOB) [28, 29]. Paired samples collected at cohort follow-up start and end from 223 children were included in the present study (median age 1.8, IQR 1.3–2.5 years).

### Antibody measurement

Total IgG was measured using an enzyme-linked immunosorbent assay (ELISA). Nunc 96-well plates (Thermo Scientific) were coated with GPI conjugated to BSA or BSA alone diluted to 10 ng/well in phosphate-buffered saline (PBS) pH 7.2, and incubated overnight at 4 °C. The next day, the plates were washed 3 times in PBS and blocked with PBS + 5% milk for 1 h at 37 °C. Plates were then washed 3 times in PBS + 0.05% Tween-20, and plasma samples from PNG children and controls diluted 1:125 in PBS + 1% milk + 0.05% Tween-20 were assayed in duplicate, with incubation overnight at 4 °C. On the third day, plates were washed 5 times in PBS + 0.05% Tween-20 and the secondary antibody horseradish peroxidase-conjugated mouse anti-human IgG (Southern biotech) diluted 100 ng/well in PBS + 1% milk + 0.05% Tween-20 was added, followed by incubation for 2 h at room temperature. Finally, plates were washed 5 times in PBS + 0.05% tween and TMB peroxidase substrate (KPL) added and incubated for 1 min and 30 s until colour developed. 1 M phosphoric acid (Sigma) was used to stop the reaction and absorbance was read at 450 nm. Plasma from seven Australian adults, and a serial dilution of a plasma pool from hyper-immune PNG adults were included as negative and positive controls, respectively. Paired samples from the same individual collected at study start and end were run on the same plate.

### Statistical analysis

Background values due to reactivity to BSA were subtracted and duplicate wells averaged. Associations with parasite density were determined using Spearman’s rank correlation. Optical density (OD) values were log_10_-transformed and differences in mean antibody levels by age, infection status, and between samples collected at start and end of follow-up were assessed using ANOVA or 2-tailed *t* tests (paired when necessary). Negative binomial generalized estimating equation (GEE) models with exchangeable correlation structure and semi-robust variance estimator were used to analyze the relationship between antibodies to *Pf*GPI and prospective risk of *P. falciparum* and *P. vivax* episodes (defined as axillary temperature ≥ 37.5 °C or history of fever in the preceding 48 h with a concurrent parasitaemia > 2500 and > 500 *P. falciparum* and *P. vivax*/μL, respectively) over the 16 months of follow-up [11]. In order to investigate this, antibody levels were classified into tertiles (cut-off values are given on Table 1), and analyses carried out comparing the incidence rate ratio (IRR) of clinical malaria in those with medium and high antibody levels versus low. Children were considered at risk from the first day after the blood sample for active follow-up was taken. For each child, the molFOB was calculated as the number of new blood-stage genetically distinct *P. falciparum* or *P. vivax* clones acquired/year-at-risk, and square-root transformed for a better fit [28, 29]. Adjustments were made for seasonal trends, village of residency, age, haemoglobin levels, Gerbich blood type, and molFOB. All analyses were performed using STATA version 12 (StataCorp).

**Table 1:** Seroprevalence of IgG antibodies to *Pf*GPI in Papua New Guinean children

	Study start	Study end	P value
	IgG level^a^ in children (% of adult levels)		
Geometric mean 95% CI	0.096 (13.87)	0.108 (15.62)	0.11
	0.083 (11.99)	0.093 (13.44)	
	0.110 (15.90)	0.127 (18.35)	
Cut-off^a^ low antibody group	0.061 (8.75)		
Cut-off^a^ medium antibody group	0.135 (19.53)		

	Study start	Study end	
	Prevalence in children (%)		
% of adult levels (cut-off^a^)			
1% (0.007)	223 (100)	223 (100)	
5% (0.035)	200 (89.67)	196 (87.89)	0.55
10% (0.069)	133 (59.64)	144 (64.57)	0.28
25% (0.173)	55 (24.66)	71 (31.84)	0.09
50% (0.346)	26 (11.66)	36 (16.14)	0.17

P values from paired 2-tailed t tests or Chi squared tests. P < 0.05 were deemed statistically significant

*95% CI* 95% confidence interval

^a^Optical density at 450 nm

## Results

### IgG antibodies to *Pf*GPI in young PNG children

IgG seroprevalence to *Pf*GPI was relatively low at the study start. It was assumed that the pooled serum from immune PNG adults represented the highest antibody levels to *Pf*GPI achievable under natural exposure and, therefore, by comparison with IgG levels observed in PNG children, the number of children that had already achieved IgG levels that were > 50, > 25 or > 10% of the maximum adult levels (Table 1) was determined. At this time point, only 11.7 and 59.6% of the PNG children had acquired IgG levels that were > 50 and > 10% of the immune adult levels (Table 1).

Overall, although the 1–3 years old children tested in this study had acquired low levels of antibodies to *Pf*GPI, the response observed was directed and significantly higher to *Pf*GPI (mean OD to GPI after BSA subtraction = 0.18, 95% CI 0.14–0.21) than to the BSA tag alone (mean OD to BSA alone = 0.08, 95% CI 0.07–0.09, P = 0.009) (Additional file 1).

### Influence of age and exposure to malaria parasites

At study start, there was no association between age and IgG levels to *Pf*GPI (P = 0.53) (Table 2). An increase in IgG levels with age was only observed in children > 24 months old and free of *P. falciparum* infection (detected by PCR) at the moment of sample collection (P = 0.009), suggesting that antibodies to *Pf*GPI are reflective of recent malaria infections. Children with a current *P. falciparum* (P = 0.084, n = 112) (although only moderately), *P. vivax* (P = 0.036, n = 125) or mixed infection (*P. falciparum* + *P. vivax*) (P = 0.004, n = 65) had higher antibody levels than children infection-free (Table 2). There were however, no associations between IgG levels and *P. falciparum* or *P. vivax* parasite densities (rho = 0.09, P > 0.18). Children with the highest IgG levels were also more likely to have a *P. falciparum* (Odds ratio [OR] 2.77, 95% CI 1.43–5.38, P = 0.003) or *P. vivax* (OR 1.91, 95% CI 0.99–3.70, P = 0.055) infection in the following 2 months.

**Table 2:** Influence of age and exposure on antibody levels to *Pf*GPI in Papua New Guinean children

	*P. falciparum*			*P. vivax*		
	n	Geom mean (95% CI)*	P value	n	Geom mean (95% CI)*	P value
Age (months)						
All children						
12–17	81	0.091 (0.074–0.111)	0.53			
18–23	49	0.083 (0.061–0.114)				
24–29	38	0.117 (0.080–0.170)				
30–35	45	0.108 (0.075–0.155)				
36–41	10	0.081 (0.032–0.204)				
PCR−						
12–17	55	0.076 (0.060–0.097)	*0.009*	37	0.067 (0.051–0.087)	0.221
18–23	22	0.053 (0.036–0.078)		19	0.074 (0.043–0.128)	
24–29	14	0.160 (0.079–0.323)		18	0.126 (0.086–0.185)	
30–35	16	0.113 (0.062–0.207)		19	0.083 (0.047–0.146)	
36–41	4	0.155 (0.014–1.737)		5	0.087 (0.049–0.156)	
PCR+						
12–17	26	0.131 (0.090–0.192)	0.43	44	0.118 (0.088–0.157)	0.69
18–23	27	0.120 (0.078–0.186)		30	0.090 (0.060–0.133)	
24–29	24	0.097 (0.062–0.153)		20	0.109 (0.056–0.210)	
30–35	29	0.106 (0.065–0.170)		26	0.131 (0.080–0.215)	
36–41	6	0.053 (0.018–0.154)		5	0.075 (0.007–0.773)	
Infection status						
PCR−	111	0.085 (0.070–0.103)	*0.084*	98	0.081 (0.067–0.098)	*0.036*
PCR+	112	0.108 (0.088–0.133)		125	0.109 (0.089–0.134)	
Infection free	51	0.061 (0.047–0.077)	*0.004*			
Pf and Pv co-infected	65	0.107 (0.079–0.144)				

*Geom mean,* geometric mean; *n*, number; *95% CI*, 95% confidence interval; *Pf*, *Plasmodium falciparum*; *Pv*, *Plasmodium vivax*

* Optical density at 450 nm. IgG levels were log10 transformed and P values calculated using two sample t tests or ANOVA. P < 0.05 were considered significant

Due to the small age range in this cohort, however, the number of genetically distinct blood-stage parasites that each child acquired over time (i.e. the molFOB) is a better proxy for exposure to malaria than age alone [28, 29]. Thus, calculating life-time exposure as a product of age and molFOB, an increase in IgG levels to *Pf*GPI was found with increasing life-time exposure to *P. vivax* blood-stage infections (Spearman’s rho = 0.15, P = 0.026), with stronger effects observed in children free of *P. vivax* infections at sample collection (rho = 0.23, P = 0.026).

The risk of malaria infection was heterogeneously distributed across the different villages where the study was conducted [30]. Anti-*Pf*GPI antibody levels did reflect such differences, and IgG levels were significantly different when grouping individuals by village of residence (P = 0.025) (Additional file 2). Individuals living in the villages Ilaita 2 and 6 (P = 0.06–0.007, n = 10 and 12, respectively), and Sunuhu 1 (P = 0.004, n = 36) had higher IgG levels to *Pf*GPI. These regional differences were significant if children were co-infected (P = 0.048) or infected with *P. vivax* (P = 0.001), but not in the absence of infection (P > 0.3) (Additional file 2). Similar differences in antibody levels to *P. falciparum* AMA1 and MSP2 within these regions have been described [7].

### Anti-*Pf*GPI antibodies and morbidity

**Fig. 1:**
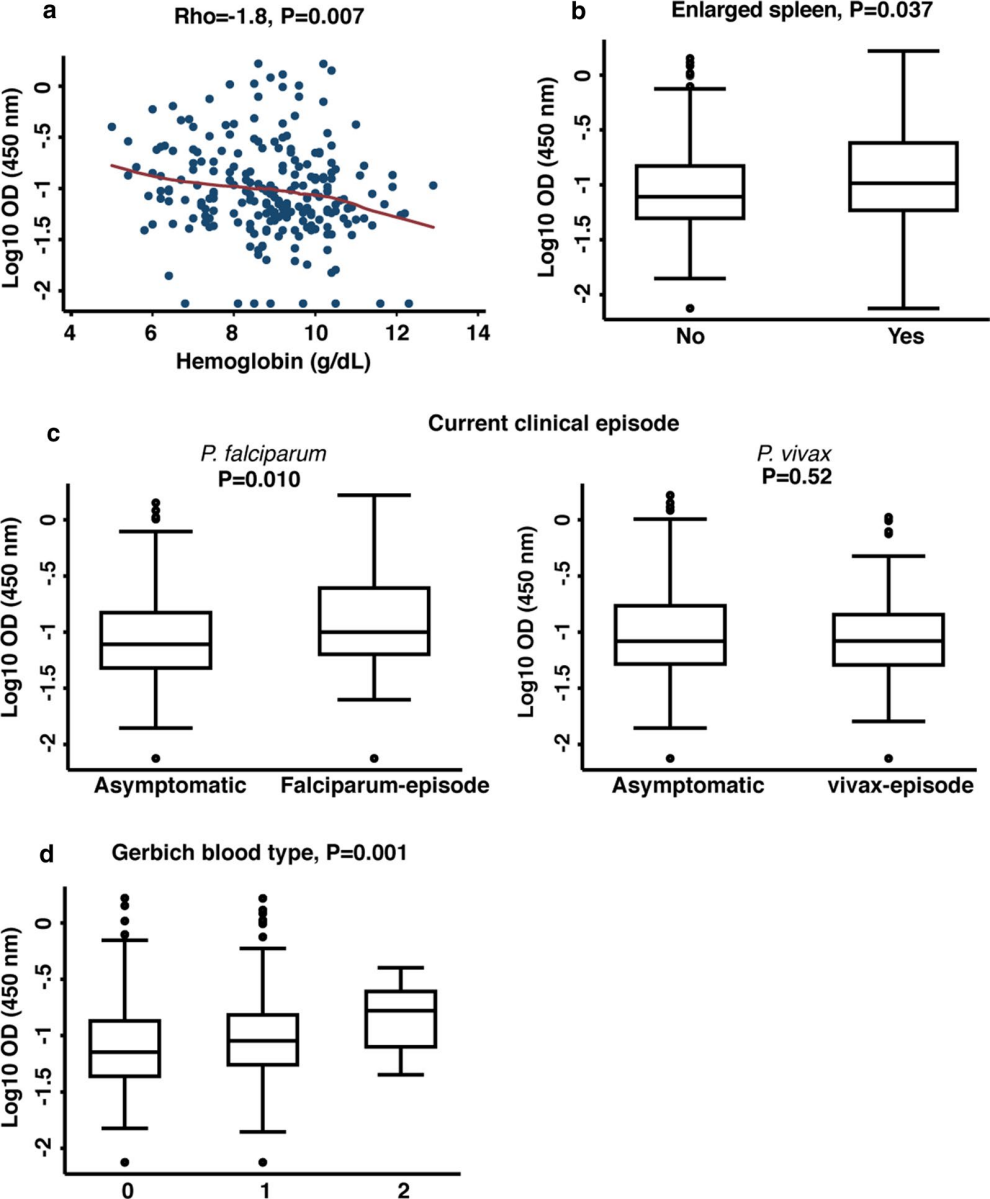
IgG to *Pf*GPI and clinical symptoms in Papua New Guinean children. **a** Scatterplot of total IgG levels (optical density at 450 nm) versus haemoglobin levels (g/dL) (n = 223) with a Lowess smoothed fitted curve. P values and rho are from Spearman’s rank correlation. Box plots show median IgG levels (black bar), minimum and maximum (whiskers) and outliers (open circles) by **b** presence of enlarged spleen (n = 59); **c** current clinical episode of any density by *P. falciparum* (n = 65) or *P. vivax* (n = 70); **d** Gerbich blood type 1 = wild-type (n = 83), 2 = heterozygote (n = 111), 3 = homozygote (n = 29). P values are from ANOVA or 2 sample t tests

Anti-*Pf*GPI antibodies were strongly associated with morbidity. IgG levels were negatively correlated with haemoglobin levels (rho = − 0.18, P = 0.007), (Fig. 1a), and elevated IgG levels were present in those with a palpable, enlarged spleen (P = 0.037) (Fig. 1b). Similarly, children with a current clinical episode by *P. falciparum* (P = 0.010), but not *P. vivax* (P = 0.21), had higher IgG levels than uninfected or asymptomatically infected children (Fig. 1c). Interestingly, there was a strong association between anti-*Pf*GPI antibodies and the children’s Gerbich blood type. The few Gerbich homozygote children had the highest IgG levels (P = 0.001) (Fig. 1d).

### IgG antibodies to *Pf*GPI and prospective risk of falciparum-malaria

**Fig. 2:**
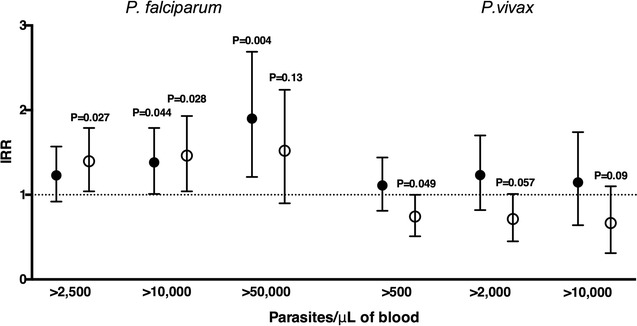
IgG to *Pf*GPI and risk of *falciparum* and vivax-malaria in Papua New Guinean children. Data are plotted as incidence rate ratios and 95% confidence intervals over 16 months of follow-up, adjusted for age, season, village of residency, haemoglobin levels and Gerbich blood type (n = 223). Black and white circles represent children with medium and high antibody levels, respectively. Clinical malaria was defined as axillary temperature ≥ 37.5 °C or history of fever in the preceding 48 h with a current *P. falciparum* parasitemia of > 2500 (n = 383); > 10,000 (n = 315); and > 50,000 parasites/μL (n = 175); and *P. vivax* > 500 (n = 301); > 2000 (n = 207); and > 10,000 parasites/μL (n = 90). IRR, 95% confidence intervals and P values are from negative binomial GEE models

Over the 16 months of follow-up, each child in the subgroup tested had an average of 1.54 (95% CI 1.38–1.73) *P. falciparum* clinical episodes with > 2500 parasites/μL/year-at-risk. Following adjustment for age, seasonal and spatial differences in malaria transmission, Gerbich blood type and haemoglobin levels, high levels of IgG to *Pf*GPI were associated with an increased risk of having *P. falciparum*-malaria (Incidence rate ratio for high versus low group [IRR_H_] 1.36, P = 0.027) (Fig. 2; Additional file 3). The risk increased for clinical episodes with higher parasite densities: > 10,000 parasites/μL (IRR_M_ 1.35, P = 0.044 and IRR_H_ 1.42, P = 0.028), > 50,000 parasites/μL (IRR_M_ 1.80, P = 0.004) (Fig. 2; Additional file 3). Once adjusted for individual differences in exposure to *P. falciparum* infections (molFOB), the strength and significance of the associations were decreased or became no longer significant (> 2500: IRR_H_ 1.26, P = 0.07; > 10,000: IRR_M_ 1.17, and IRR_H_ 1.29, P = 0.08–0.26; > 50,000: IRR_M_ 1.59, P = 0.018) (Additional file 3), indicating that in this age group, high IgG levels to *Pf*GPI are markers of children who had higher exposure to *P. falciparum* parasites, which consequently led to a higher risk of having clinical disease.

### IgG antibodies to *Pf*GPI and prospective risk of vivax-malaria

The average of clinical episodes caused by *P. vivax* (> 500 parasites/μL) was 1.22 (95% CI 1.05–1.42) per year-at-risk. In contrast to that observed for *P. falciparum*-malaria, after adjustments for confounders and differences in individual exposure to *P. vivax* blood-stage infections, high IgG levels to *Pf*GPI were associated with a modestly reduced risk of vivax-malaria (IRR_H_ 0.72, P = 0.049) (Fig. 2; Additional file 3). As for *P. falciparum*, the associations with protection tended to be stronger for clinical episodes with higher parasite density, although not of statistical significance given the reduced power (> 2000 parasites/μL IRR_H_ 0.68, P = 0.057; > 10,000 parasites/μL IRR_H_ 0.59, P = 0.094) (Fig. 2; Additional file 3). These results indicate that high levels of IgG to *Pf*GPI in this age group are also markers of acquired immunity to *P. vivax*.

### Antibodies to *Pf*GPI after 16 months

At the end of the 16 months of follow-up, 64.6 and 16.1% of the children had reached IgG levels that were > 10 and > 50% of the IgG levels observed in the adult immune pool (Table 1). Although slightly higher, antibody levels were very similar (rho = 0.57, P < 0.001) and this seroprevalence was not statistically different than the observed at the study start (P > 0.17), suggesting that in this age group, anti-*Pf*GPI antibodies are short-lived or unstable.

At the end of the study, there was no association between IgG levels and age (P > 0.18), life-time exposure (P > 0.20), *P. falciparum* or *P. vivax* infection status (P > 0.05) (Additional file 4). No difference in IgG levels was observed between children who experienced a clinical episode in the last 2 months and those who did not (P > 0.18), or by the number of clinical episodes that each child had over the follow-up period for *P. falciparum* or *P. vivax* (P > 0.31) (Additional file 4). Similarly, there was no difference in IgG levels at the end of the follow-up between children who did and who did not experience severe malaria during follow-up (n = 24, P = 0.97).

At the end of the study, the only factor associated with differences in antibody levels was village of residency for those currently co-infected (P = 0.046) or with a *P. vivax* infection (P = 0.008) (Additional file 2).

## Discussion

A better understanding of the acquisition of immunity to malaria parasites in different age groups and transmission settings is essential for the identification of antigens useful as biomarkers of exposure/immunity, or with potential for vaccine development—especially for *P. vivax,* since a continuous in vitro culture system is still inexistent [4, 5]. In the present study, antibody levels to a synthetic glycan correspondent to *Pf*GPI [31] was measured in a cohort of children 1–3 years old from PNG [30], exploring the associations between antibody levels and risk of *P. falciparum* and *P. vivax*-malaria.

Despite the very high transmission intensity in East Sepik Province when the cohort study was conducted [30], seroprevalence of antibodies to *Pf*GPI was low in this age group. Similar low seroprevalence have been described in children < 6 years from Madang Province in PNG [32], as well as in Indonesia [33], Kenya [18] and Gambia [34]. One explanation for this is the low ability of the immune system of very young children (< 2 years old) in producing antibodies against carbohydrate antigens [35]. This also suggests that the majority of GPI that the immune system has access to and thus can produce antibodies to is the free form, rather than the form that anchors proteins to the parasite membrane. If physically attached to their GPI anchors, parasite surface proteins might be expected to provide T cell help for anti-GPI antibody production [15]. Although not observed in the young children included in the study, seroprevalence and magnitude of antibody responses to *Pf*GPI have been described to increase with age and decline with parasite density in PNG [32] and Kenyan adults [18].

For the young children included in this study, recent *P. falciparum* and *P. vivax* infections were the main determinant of antibody levels to GPI. The rapid although transient peaks in antibody levels in the presence of a current infection might suggest that they are generated by the differentiation of naive B-cells into short-lived plasma cells driven by the concurrent infection rather than by long-lived plasma cells generated from previous infections, as previously described for malarial protein antigens [36]. Given the absence of peptide epitopes for conventional T cells, antibodies to free GPI are likely to be T cell-independent during the first malaria infections [15]. Although they can stimulate antigen-specific B cells, memory is not generated, and accessory cells (e.g. macrophages and dendritic cells) and co-stimulatory signals (e.g. IL-1) are thus required for an effective immune response [37]. Later with increasing exposure, or if attached to an immunogenic carrier, however, GPI might be taken up by follicular B cells, be processed and presented on cell surface major histocompatibility complex class II (MHCII) molecules, where they may engage peptide-specific T cells [15, 35]. Memory B cells can thus be generated during this T cell-dependent process, and be re-activated upon future stimulation [35].

Children with homozygote Gerbich blood type (Gerbich negative) had higher antibody levels to *PfGPI* than heterozygote or wild type children. The Gerbich antigen is expressed on glycophorins C (GPC) and D (GPD) [38], and both GPC/D interact with the 4.1 R protein complex and contribute to the stability of the erythrocyte membrane [38, 39]. A high incidence of Gerbich negative in PNG been hypothesized as an advantage against infection and severe malaria [40, 41]. While it was found that deletion of the exon 3 result in Gerbich negativity and make *P. falciparum* unable to invade erythrocytes using the erythrocyte binding protein 140 [EBA140] [39, 42], to date, clinical studies have not been able to show a consistent association between risk of malaria and this phenotype [43–45]. Further in-depth studies will be required to elucidate whether the interaction between Gerbich genotype, reduced parasite invasion and slower parasite growth result in increased host immune-responses (including to *Pf*GPI), and whether this may indeed combine to provide protection against *P. falciparum* or *P. vivax* malaria [46].

In young PNG children, high antibody levels to *Pf*GPI were associated with higher risk of *P. falciparum* malaria. In contrast, they were also associated with reduced risk of *P. vivax* malaria. This accurately reflects the different levels of naturally acquired immunity to the two species in this cohort: while in these children incidence of *P. vivax* episodes significantly decreases starting in the 2nd year of life, the burden of *P. falciparum* infection continues to increase until the 4th year of life [30]. This difference is related to a significantly higher exposure to *P. vivax* than *P. falciparum* blood-stage infections, i.e. *P. vivax* molFOB was considerably higher than *P. falciparum* molFOB (14 versus 5.5 parasite clones/child/year-at-risk, respectively). This high number of *P. vivax* clones that infect children in early childhood thus contribute to a very rapid acquisition of immunity to clinical *P. vivax* malaria, not yet reached for *P. falciparum* [29, 30]. Acquisition of immunity to *P. falciparum* in high transmission settings such as PNG is achieved a number of years later (~ 10 years old) with increasing exposure to *P. falciparum* infections [7, 8]. Anti-*Pf*GPI antibodies in this age group seem to be an accurate reflection of the children’s current immune-status to both *P. falciparum* and *P. vivax* malaria, acting as both a biomarker of increased risk of *P. falciparum,* able to identify individuals with the highest level of exposure to *P. falciparum* recent infections, as well as a biomarker of acquired immunity to *P. vivax.*

In 2002, a study in rodent models firstly showed that antibodies raised against *Pf*GPI were able to delay mortality by *Plasmodium berghei*, demonstrating proof of concept for a GPI-based anti-toxic malaria vaccine [31]. The antagonists of GPI-mediated signaling and murine monoclonal antibodies against *Pf*GPIs were shown to be able to block the induction of toxic responses, also suggesting that GPI-based therapy is possible [47, 48]. In more recent studies, GPI was found to be present across all stages of the malaria parasites life cycles. Furthermore, in a pre-clinical evaluation of a GPI-based vaccine in *P. berghei* models, the vaccine showed efficacy in sporozoite challenges, was able to reduce parasite replication and transmission to mosquitoes (unpublished data, Schofield.) Altogether, these findings suggest that a GPI vaccine may be able to prevent both blood-stage and liver infections, disease and block transmission of parasite from human to mosquito, thus acting as a unique carbohydrate multi-stage, multi-parasite vaccine. Consistent with this, high levels of the anti-GPI antibodies have been correlated with resistance to clinical symptoms, such as anaemia and fever [18], and lower levels observed among Senegalese adults with cerebral malaria compared to individuals with uncomplicated malaria [49]. Although anti-*Pf*GPI antibodies are short-lived or intermittent in very young children, older children and adults seem to be able to sustain high antibody levels for longer [18, 32–34, 50]. Furthermore, GPI low immunogenicity in young children and can be overcome if the antigen is conjugated to a protein carrier, which can also help stimulation of B-cell memory formation [35]. Future functional studies are now necessary to confirm whether anti-*Pf*GPI antibodies contribute to the protection observed against *P. vivax*, or only act as a mirror of the protection conferred by antibodies to other antigenic targets.

This study highlights anti-*Pf*GPI antibodies as a possible biomarker of anti-malaria immunity in very young children. Further studies including older age groups will confirm its utility as a biomarker of immunity for *P. vivax*, and whether they will indeed also reflect acquired immunity to *P. falciparum*.

## Conclusions

The findings of this study highlight IgG to *Pf*GPI as potentially useful serological biomarkers of immune-status in young children to help malaria control programs identify populations at risk. Additional studies including older age groups will confirm the utility of these responses as a biomarker of immunity to *P. vivax*, and whether they will indeed also reflect acquired immunity to *P. falciparum*. Future functional studies are also necessary to confirm whether anti-*Pf*GPI antibodies contribute to the protection observed against *P. vivax*, or only act as a mirror of the protection conferred by antibodies to other antigenic targets.

## Additional files

**Additional file 1.** Antibody responses to *Pf*GPI and the BSA tag in 1-3 years old children. Box plots show median IgG levels (central bar), minimum and maximum (whiskers) and outliers (grey circles). n=223. P value is from t-test.

**Additional file 2.** IgG to *Pf*GPI by village of residency.

**Additional file 3.** Association between IgG to *Pf*GPI and protection against clinical malaria in Papua New Guinean children.

**Additional file 4.** Influence of age and exposure on antibody levels to *Pf*GPI in Papua New Guinean children.
