# Supplementary figures and images for: IgG antibodies to synthetic GPI are biomarkers of immune-status to both Plasmodium falciparum and Plasmodium vivax malaria in young children
Source: Malar J. 2017 Sep 25;16:386. doi: 10.1186/s12936-017-2042-2 (PMC5613389; doi:10.1186/s12936-017-2042-2)

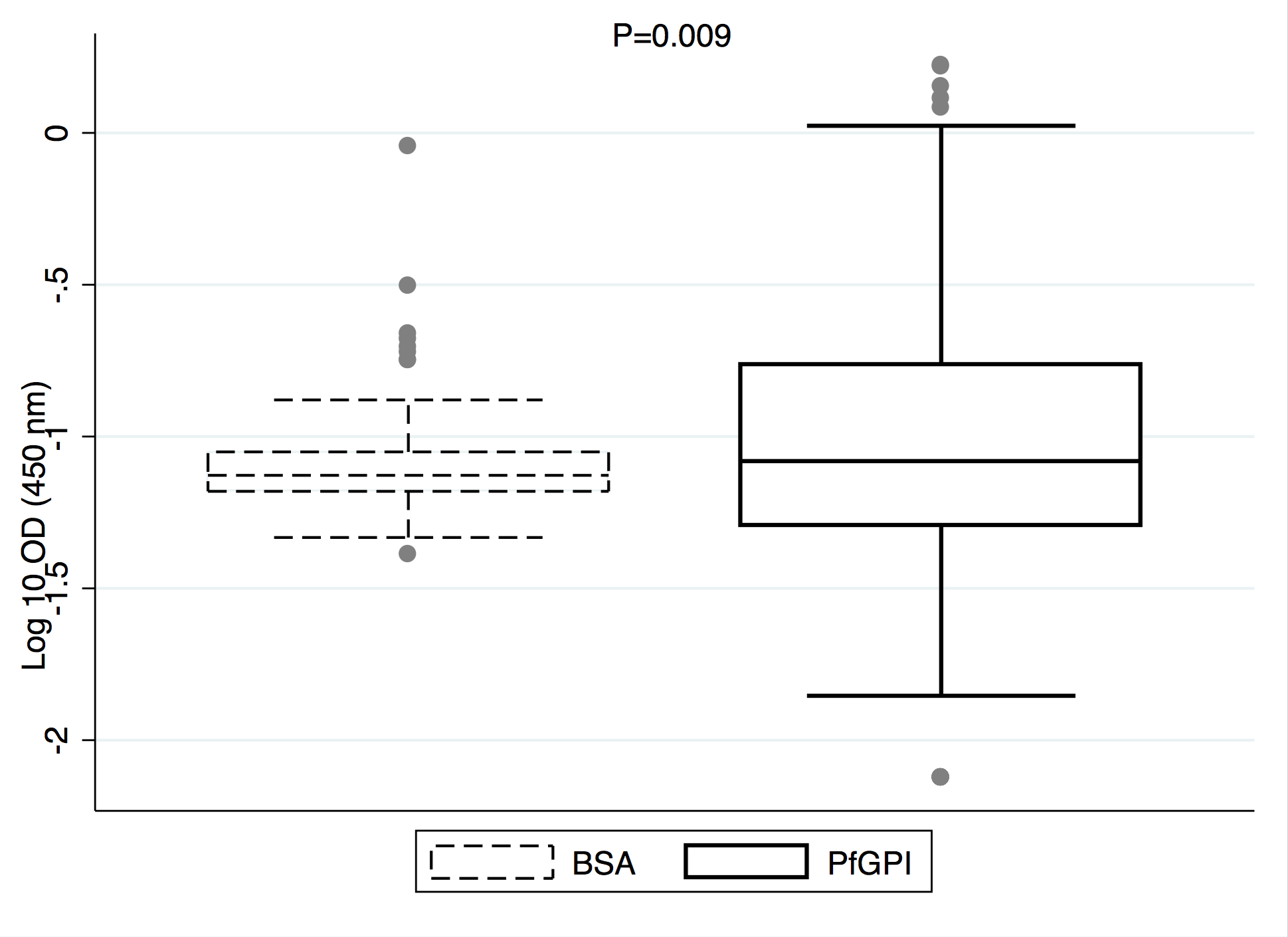

Supplement: Supplementary file 1 — Additional file 1. Antibody responses to PfGPI and the BSA tag in 1-3 years old children. Box plots show median IgG levels (central bar), minimum and maximum (whiskers) and outliers (grey circles). n=223. P value is from t-test. [file 12936_2017_2042_MOESM1_ESM.tif]
