# Supplementary material for: IgG antibodies to synthetic GPI are biomarkers of immune-status to both Plasmodium falciparum and Plasmodium vivax malaria in young children
Source: Malar J. 2017 Sep 25;16:386. doi: 10.1186/s12936-017-2042-2 (PMC5613389; doi:10.1186/s12936-017-2042-2)
Supplement: Supplementary file 2 — Additional file 2. IgG to PfGPI by village of residency. [file 12936_2017_2042_MOESM2_ESM.docx]

**Additional file 2: IgG to *Pf*GPI by village of residency.**

|  | **Study start** | | | | | | **Study end** | | | | | |
| --- | --- | --- | --- | --- | --- | --- | --- | --- | --- | --- | --- | --- |
|  | ***P. falciparum*** | | | ***P. vivax*** | | | ***P. falciparum*** | | | ***P. vivax*** | | |
| **Village** | **n** | **Geom mean**  **(95% CI)*** | **P value** | **n** | **Geom mean**  **(95% CI)*** | **P value** | **n** | **Geom mean**  **(95% CI)*** | **P value** | **n** | **Geom mean**  **(95% CI)*** | **P value** |
| **All children** |  |  |  |  |  |  |  |  |  |  |  |  |
| Ilaita 1 | 18 | 0.061 (0.036-0.102) | **0.025** |  |  |  | 18 | 0.069 (0.036-0.132) | 0.10 | 5 |  |  |
| Ilaita 2 | 10 | 0.187 (0.078-0.447) |  |  |  |  | 10 | 0.170 (0.080-0.359) |  | 3 |  |  |
| Ilaita 3 | 17 | 0.067 (0.047-0.096) |  |  |  |  | 17 | 0.082 (0.050-0.137) |  | 9 |  |  |
| Ilaita 4 | 23 | 0.091 (0.049-0.169) |  |  |  |  | 23 | 0.108 (0.062-0.187) |  | 7 |  |  |
| Ilaita 5 | 10 | 0.096 (0.073-0.126) |  |  |  |  | 10 | 0.113 (0.060-0.213) |  | 2 |  |  |
| Ilaita 6 | 12 | 0.126 (0.064-0.246) |  |  |  |  | 12 | 0.177 (0.112-0.279) |  | 5 |  |  |
| Ilaita 7 | 21 | 0.093 (0.054-0.160) |  |  |  |  | 21 | 0.139 (0.079-0.242) |  | 9 |  |  |
| Ingambils | 28 | 0.089 (0.061-0.129) |  |  |  |  | 28 | 0.085 (0.050-0.146) |  | 7 |  |  |
| Kamakor | 20 | 0.059 (0.036-0.099) |  |  |  |  | 20 | 0.067 (0.041-0.110) |  | 6 |  |  |
| Sunuhu 1 | 36 | 0.148 (0.110-0.197) |  |  |  |  | 36 | 0.158 (0.107-0.235) |  | 12 |  |  |
| Sunuhu 2 | 28 | 0.104 (0.069-0.158) |  |  |  |  | 28 | 0.107 (0.067-0.171) |  | 3 |  |  |
| **PCR -** |  |  |  |  |  |  |  |  |  |  |  |  |
| Ilaita 1 | 9 | 0.047 (0.023-0.097) | 0.35 | 7 | 0.051 (0.020-0.133) | 0.49 | 9 | 0.069 (0.031-0.151) | 0.39 | 13 | 0.125 (0.025-0.616) | 0.26 |
| Ilaita 2 | 9 | 0.198 (0.074-0.530) |  | 5 | 0.080 (0.047-0.138) |  | 8 | 0.187 (0.073-0.480) |  | 7 | 0.131 (0.014-1.190) |  |
| Ilaita 3 | 10 | 0.068 (0.039-0.118) |  | 11 | 0.059 (0.040-0.088) |  | 11 | 0.119 (0.067-0.210) |  | 8 | 0.068 (0.029-0.162) |  |
| Ilaita 4 | 17 | 0.077 (0.040-0.150) |  | 14 | 0.080 (0.034-0.184) |  | 17 | 0.093 (0.048-0.181) |  | 16 | 0.087 (0.019-0.386) |  |
| Ilaita 5 | 4 | 0.094 (0.048-0.185) |  | 3 | 0.079 (0.029-0.213) |  | 6 | 0.114 (0.039-0.331) |  | 8 | 0.172 (0.014-2.197) |  |
| Ilaita 6 | 10 | 0.112 (0.050-0.249) |  | 8 | 0.088 (0.042-0.185) |  | 7 | 0.169 (0.077-0.369) |  | 7 | 0.174 (0.053-0.578) |  |
| Ilaita 7 | 16 | 0.086 (0.042-0.175) |  | 15 | 0.062 (0.038-0.100) |  | 16 | 0.125 (0.061-0.256) |  | 12 | 0.083 (0.043-0.161) |  |
| Ingambils | 18 | 0.071 (0.050-0.101) |  | 10 | 0.122 (0.067-0.222) |  | 15 | 0.065 (0.0270-0.156) |  | 21 | 0.075 (0.024-0.235) |  |
| Kamakor | 5 | 0.084 (0.035-0.201) |  | 5 | 0.114 (0.0183-0.716) |  | 8 | 0.091 (0.044-0.188) |  | 14 | 0.060 (0.035-0.103) |  |
| Sunuhu 1 | 5 | 0.095 (0.045-0.198) |  | 10 | 0.135 (0.080-0.228) |  | 8 | 0.087 (0.023-0.330) |  | 24 | 0.210 (0.108-0.409) |  |
| Sunuhu 2 | 8 | 0.090 (0.054-0.151) |  | 10 | 0.076 (0.046-0.125) |  | 12 | 0.208 (0.103-0.419) |  | 25 | 0.043 (0.006-0.280) |  |
| **PCR +** |  |  |  |  |  |  |  |  |  |  |  |  |
| Ilaita 1 | 9 | 0.078 (0.033-0.185) | 0.17 | 11 | 0.068 (0.033-0.140) | **0.001** | 9 | 0.069 ( 0.020-0.237) | **0.008** |  | 0.055 (0.025-0.119) | 0.21 |
| Ilaita 2 | 1 | 0.1105 |  | 5 | 0.435 (0.102-1.863) |  | 2 | 0.115 (<0.001-121.651) |  |  | 0.190 (0.065-0.551) |  |
| Ilaita 3 | 7 | 0.066 (0.035-0.122) |  | 6 | 0.084 (0.032-0.220) |  | 6 | 0.042 (0.016-0.109) |  |  | 0.105 (0.051-0.202) |  |
| Ilaita 4 | 4 | 0.148 (0.022-0.976) |  | 9 | 0.113 (0.037-0.346) |  | 6 | 0.162 (0.043-0.612) |  |  | 0.118 (0.064-0.219) |  |
| Ilaita 5 | 6 | 0.098 (0.065-0.147) |  | 7 | 0.105 (0.075-0.147) |  | 4 | 0.111 (0.031-0.395) |  |  | 0.102 (0.045-0.228) |  |
| Ilaita 6 | 2 | 0.222 (0.001-44.618) |  | 4 | 0.255 (0.042-1.560) |  | 5 | 0.189 (0.091-0.391) |  |  | 0.179 (0.106-0.303) |  |
| Ilaita 7 | 5 | 0.118 (0.047-0.294) |  | 6 | 0.253 (0.057-1.115) |  | 5 | 0.195 (0.085-0.448) |  |  | 0.204 (0.087-0.479) |  |
| Ingambils | 10 | 0.132 (0.054-0.322) |  | 18 | 0.074 (0.045-0.122) |  | 13 | 0.117 (0.062-0.223) |  |  | 0.089 (0.046-0.172) |  |
| Kamakor | 15 | 0.053 (0.027-0.102) |  | 15 | 0.048 (0.029-0.078) |  | 12 | 0.055 (0.026-0.115) |  |  | 0.070 (0.034-0.144) |  |
| Sunuhu 1 | 31 | 0.159 (0.115-0.219) |  | 26 | 0.153 (0.106-0.221) |  | 28 | 0.188 (0.128-0.274) |  |  | 0.137 (0.082-0.229) |  |
| Sunuhu 2 | 20 | 0.110 (0.063-0.195) |  | 18 | 0.125 (0.068-0.228) |  | 16 | 0.065 (0.038-0.114) |  |  | 0.120 (0.073-0.197) |  |
| **Infection free** |  |  |  |  |  |  |  |  |  |  |  |  |
| Ilaita 1 | 3 | 0.030 (0.001-1.027) | 0.79 |  |  |  | 1 | 0.063 | 0.11 |  |  |  |
| Ilaita 2 | 5 | 0.080 (0.047-0.138) |  |  |  |  | 3 | 0.131 (0.014-1.190) |  |  |  |  |
| Ilaita 3 | 8 | 0.054 (0.033-0.090) |  |  |  |  | 6 | 0.105 (0.044-0.256) |  |  |  |  |
| Ilaita 4 | 9 | 0.068 (0.025-0.187) |  |  |  |  | 5 | 0.038 (0.013-0.103) |  |  |  |  |
| Ilaita 5 | 1 | 0.055 |  |  |  |  | 2 | 0.172 (0.014-2.197) |  |  |  |  |
| Ilaita 6 | 7 | 0.082 (0.035-0.194) |  |  |  |  | 5 | 0.174 (0.053-0.578) |  |  |  |  |
| Ilaita 7 | 10 | 0.045 (0.026-0.078) |  |  |  |  | 7 | 0.070 (0.032-0.151) |  |  |  |  |
| Ingambils | 5 | 0.080 (0.043-0.150) |  |  |  |  | 1 | 0.0305 |  |  |  |  |
| Kamakor | 0 |  |  |  |  |  | 2 | 0.080 (0.002-4.086) |  |  |  |  |
| Sunuhu 1 | 2 | 0.079 (0.002-4.109) |  |  |  |  | 1 | 0.588 |  |  |  |  |
| Sunuhu 2 | 1 | 0.038 |  |  |  |  | 1 | 0.102 |  |  |  |  |
| **Pf & Pv co-infected** |  |  |  |  |  |  |  |  |  |  |  |  |
| Ilaita 1 | 5 | 0.080 (0.012-0.525) | **0.048** |  |  |  | 5 | 0.037 (0.005-0.278) | **0.046** |  |  |  |
| Ilaita 2 | 1 | 0.111 |  |  |  |  | 2 | 0.115 (<0.001-121.651) |  |  |  |  |
| Ilaita 3 | 4 | 0.059 (0.016-0.221) |  |  |  |  | 3 | 0.062 (0.034-0.111) |  |  |  |  |
| Ilaita 4 | 1 | 0.789 |  |  |  |  | 4 | 0.078 (0.036-0.172) |  |  |  |  |
| Ilaita 5 | 4 | 0.100 (0.048-0.205) |  |  |  |  | 4 | 0.111 (0.031-0.395) |  |  |  |  |
| Ilaita 6 | 1 | 0.337 |  |  |  |  | 5 | 0.189 (0.091-0.391) |  |  |  |  |
| Ilaita 7 | 0 |  |  |  |  |  | 2 | 0.230 (0.049-1.088) |  |  |  |  |
| Ingambils | 5 | 0.094 (0.014-0.624) |  |  |  |  | 7 | 0.150 (0.067-0.335) |  |  |  |  |
| Kamakor | 10 | 0.036 (0.019-0.067) |  |  |  |  | 8 | 0.057 (0.017-0.185) |  |  |  |  |
| Sunuhu 1 | 23 | 0.160 (0.107-0.240) |  |  |  |  | 17 | 0.185 (0.112-0.305) |  |  |  |  |
| Sunuhu 2 | 11 | 0.141 (0.051-0.390) |  |  |  |  | 14 | 0.074 (0.040-0.136) |  |  |  |  |

Abbreviations: Geom mean = geometric mean; n = number; 95%CI = 95% confidence interval; Pf = *Plasmodium falciparum*; Pv = *Plasmodium vivax*. * Optical density at 450 nm. IgG levels were log10 transformed and P values calculated using ANOVA. P < 0.05 were considered significant.
