# Supplementary material for: IgG antibodies to synthetic GPI are biomarkers of immune-status to both Plasmodium falciparum and Plasmodium vivax malaria in young children
Source: Malar J. 2017 Sep 25;16:386. doi: 10.1186/s12936-017-2042-2 (PMC5613389; doi:10.1186/s12936-017-2042-2)
Supplement: Supplementary file 3 — Additional file 3. Association between IgG to PfGPI and protection against clinical malaria in Papua New Guinean children. [file 12936_2017_2042_MOESM3_ESM.docx]

**Additional file 3: Association between IgG to *Pf*GPI and protection against clinical malaria in Papua New Guinean children.**

|  | **Antibody level** | **uIRR** | **95%CI** | | **P value** | **aIRR** | **95%CI** | | **P value** | **aIRR molFOB** | **95%CI** | | **P value** |
| --- | --- | --- | --- | --- | --- | --- | --- | --- | --- | --- | --- | --- | --- |
| 2,000  Pf/uL | M | 1.12 | 0.85 | 1.48 | 0.42 | 1.20 | 0.92 | 1.57 | 0.17 | 1.07 | 0.84 | 1.36 | 0.61 |
|  | H | 1.22 | 0.93 | 1.62 | 0.16 | 1.36 | 1.04 | 1.79 | **0.027** | 1.26 | 0.99 | 1.61 | 0.066 |
| 10,000 Pf/uL | M | 1.18 | 0.87 | 1.61 | 0.29 | 1.35 | 1.01 | 1.79 | **0.044** | 1.17 | 0.89 | 1.53 | 0.26 |
|  | H | 1.18 | 0.85 | 1.62 | 0.32 | 1.42 | 1.04 | 1.93 | **0.028** | 1.29 | 0.97 | 1.71 | 0.079 |
| 50,000 Pf/uL | M | 1.53 | 1.05 | 2.25 | **0.028** | 1.80 | 1.21 | 2.69 | **0.004** | 1.59 | 1.08 | 2.35 | **0.018** |
|  | H | 1.09 | 0.70 | 1.70 | 0.69 | 1.42 | 0.90 | 2.24 | 0.13 | 1.30 | 0.85 | 1.98 | 0.23 |
| 500  Pv/μL | M | 0.97 | 0.68 | 1.36 | 0.84 |  |  |  |  | 1.08 | 0.81 | 1.44 | 0.61 |
|  | H | 0.78 | 0.52 | 1.16 | 0.23 |  |  |  |  | 0.72 | 0.51 | 1.00 | **0.049** |
| 2,000 Pv/μL | M | 1.00 | 0.66 | 1.52 | 0.99 |  |  |  |  | 1.18 | 0.82 | 1.70 | 0.37 |
|  | H | 0.74 | 0.45 | 1.22 | 0.24 |  |  |  |  | 0.68 | 0.45 | 1.01 | **0.057** |
| 10,000 Pv/μL | M | 0.82 | 0.48 | 1.41 | 0.47 |  |  |  |  | 1.06 | 0.64 | 1.74 | 0.83 |
|  | H | 0.53 | 0.27 | 1.01 | **0.055** |  |  |  |  | 0.59 | 0.31 | 1.10 | 0.09 |

Abbreviations: Pf = *Plasmodium falciparum*; Pv = *Plasmodium vivax*; 95%CI = 95% confidence interval; M = medium IgG levels; H = high IgG levels; uIRR = unadjusted IRR; aIRR = adjusted for age, molFOB, region, season, hemoglobin levels and Gerbich blood type; aIRR molFOB = adjusted for the same variables as aIRR as well as molFOB. uIRR, aIRR, 95% confidence intervals and P values are from GEE models. P < 0.05 were considered significant.
