# Supplementary material for: IgG antibodies to synthetic GPI are biomarkers of immune-status to both Plasmodium falciparum and Plasmodium vivax malaria in young children
Source: Malar J. 2017 Sep 25;16:386. doi: 10.1186/s12936-017-2042-2 (PMC5613389; doi:10.1186/s12936-017-2042-2)
Supplement: Supplementary file 4 — Additional file 4. Influence of age and exposure on antibody levels to PfGPI in Papua New Guinean children. [file 12936_2017_2042_MOESM4_ESM.docx]

**Additional file 4: Additional file 3: Influence of age and exposure on antibody levels to *Pf*GPI in Papua New Guinean children.**

|  |  |  | *P. falciparum* | | | | *P. vivax* | | | | |
| --- | --- | --- | --- | --- | --- | --- | --- | --- | --- | --- | --- |
|  |  | n | Geom mean (95% CI)* | P value | Rho | P value | n | Geom mean (95% CI)* | P value | Rho | P value |
| Age (months) | **All children** |  |  |  |  |  |  |  |  |  |  |
|  | 12-17 | 24 | 0.126 (0.081-0.198) | 0.18 |  |  |  |  |  |  |  |
|  | 18-23 | 47 | 0.074 (0.050-0.109) |  |  |  |  |  |  |  |  |
|  | 24-29 | 52 | 0.137 (0.104-0.180) |  |  |  |  |  |  |  |  |
|  | 30-35 | 50 | 0.114 (0.081-0.161) |  |  |  |  |  |  |  |  |
|  | 36-41 | 27 | 0.099 (0.061-0.160) |  |  |  |  |  |  |  |  |
|  | >42 | 23 | 0.119 (0.067-0.212) |  |  |  |  |  |  |  |  |
|  | **PCR -** |  |  |  |  |  |  |  |  |  |  |
|  | 12-17 | 18 | 0.134 (0.080-0.224) | 0.07 |  |  | 12 | 0.111 (0.055-0.224) | 0.87 |  |  |
|  | 18-23 | 31 | 0.067 (0.044-0.101) |  |  |  | 13 | 0.074 (0.044-0.124) |  |  |  |
|  | 24-29 | 22 | 0.141 (0.094-0.213) |  |  |  | 15 | 0.107 (0.056-0.202) |  |  |  |
|  | 30-35 | 18 | 0.128 (0.061-0.268) |  |  |  | 19 | 0.100 (0.052-0.193) |  |  |  |
|  | 36-41 | 15 | 0.085 (0.038-0.190) |  |  |  | 6 | 0.150 (0.054-0.415) |  |  |  |
|  | >42 | 13 | 0.192 (0.092-0.401) |  |  |  | 3 | 0.106 (0.014-0.792) |  |  |  |
|  | **PCR +** |  |  |  |  |  |  |  |  |  |  |
|  | 12-17 | 6 | 0.106 (0.030-0.381) | 0.62 |  |  | 12 | 0.144 (0.074-0.279) | 0.17 |  |  |
|  | 18-23 | 16 | 0.090 (0.038-0.212) |  |  |  | 34 | 0.074 (0.044-0.123) |  |  |  |
|  | 24-29 | 30 | 0.133 (0.090-0.197) |  |  |  | 37 | 0.151 (0.112-0.205) |  |  |  |
|  | 30-35 | 32 | 0.107 (0.074-0.157) |  |  |  | 31 | 0.124 (0.082-0.187) |  |  |  |
|  | 36-41 | 12 | 0.120 (0.069-0.206) |  |  |  | 21 | 0.088 (0.049-0.157) |  |  |  |
|  | >42 | 10 | 0.064 (0.026-0.158) |  |  |  | 20 | 0.121 (0.062-0.234) |  |  |  |
| Life-time exposure | **All children** | n | Geom mean (95% CI)* | P value |  |  | n | Geom mean (95% CI)* | P value |  |  |
|  | *L* | 75 | 0.094 (0.073-0.120) | 0.43 |  |  | 72 | 0.111 (0.083-0.149) |  |  |  |
|  | *M* | 72 | 0.118 (0.088-0.159) |  |  |  | 75 | 0.120 (0.093-0.155) | 0.60 |  |  |
|  | *H* | 76 | 0.115 (0.087-0.152) |  |  |  | 71 | 0.098 (0.073-0.132) |  |  |  |
|  | **PCR -** |  |  |  |  |  |  |  |  |  |  |
|  | *L* | 61 | 0.085 (0.064-0.113) | 0.20 |  |  | 42 | 0.099 (0.070-0.139) | 0.85 |  |  |
|  | *M* | 35 | 0.131 (0.083-0.207) |  |  |  | 22 | 0.113 (0.071-0.179) |  |  |  |
|  | *H* | 15 | 0.115 (0.065-0.201) |  |  |  | 29 | 0.093 (0.054-0.161) |  |  |  |
|  | **PCR +** |  |  |  |  |  |  |  |  |  |  |
|  | *L* | 14 | 0.143 (0.080-0.256) | 0.75 |  |  | 30 | 0.131 (0.076-0.224) | 0.39 |  |  |
|  | *M* | 37 | 0.107 (0.072-0.159) |  |  |  | 53 | 0.123 (0.089-0.170) |  |  |  |
|  | *H* | 61 | 0.115 (0.083-0.160) |  |  |  | 42 | 0.102 (0.072-0.144) |  |  |  |
|  |  | n | Geom mean (95% CI)* | P value |  |  | n | Geom mean (95% CI)* | P value |  |  |
| Infection status | **PCR -** | 117 | 0.110 (0.087-0.137) | 0.88 |  |  | 68 | 0.101 (0.078-0.133) | 0.58 |  |  |
|  | **PCR +** | 106 | 0.107 (0.086-0.134) |  |  |  | 155 | 0.111 (0.092-0.136) |  |  |  |
|  | **Pf & Pv no infection** | 34 | 0.092 (0.067-0.128) | 0.55 |  |  |  |  |  |  |  |
|  | **Pf & Pv co-infected** | 72 | 0.105 (0.081-0.136) |  |  |  |  |  |  |  |  |
|  |  | n | Geom mean (95% CI)* | P value |  |  | n | Geom mean (95% CI)* | P value |  |  |
| Clinical episode | **No** | 148 | 0.100 (0.084-0.120) | 0.18 |  |  | 162 | 0.106 (0.088-0.128) | 0.68 |  |  |
|  | **Yes** | 75 | 0.126 (0.093-0.172) |  |  |  | 61 | 0.114 (0.086-0.152) |  |  |  |

Abbreviations: Geom mean = geometric mean; Pf = *Plasmodium falciparum*; Pv = *Plasmodium vivax*; 95%CI = 95% confidence interval; L = Low; M = medium IgG levels; H = high IgG levels. * Optical density at 450 nm. P values were calculated using 2 sample t-tests or ANOVA. P < 0.05 were considered significant.
